# Policy Series: Interdisciplinary Public Policy Discussion

**DOI:** 10.1093/geroni/igaa057.3131

**Published:** 2020-12-16

**Authors:** Brian Lindberg, Linda Harootyan

## Abstract

Aging and health care public policy in Washington can be driven and influenced by the work of GSA researchers, educators, and practitioners from across the nation. This session will examine and explore public policy priorities from an interdisciplinary perspective and consider opportunities for communicating these policies with key policymakers. This session is an interdisciplinary look at policy issues in aging with the speakers representing views from the six sections of GSA. This session, organized by the GSA Public Policy Committee, will provide both GSA section leadership and attendees an opportunity to have an open dialogue on important public policy issues of significance in the field of aging. The session discussant will help to facilitate a robust discussion of the presentations by speakers. Organized by the GSA Public Policy Committee, this dialogue will benefit the work of the Committee in 2021.

